# Association between exercise habit changes and incident dementia after ischemic stroke

**DOI:** 10.1038/s41598-023-31229-z

**Published:** 2023-03-09

**Authors:** Dae Young Cheon, Kyung do Han, Chi-hun Kim, Mi Sun Oh, Byung-Chul Lee, Yerim Kim, Sang-Hwa Lee, Chulho Kim, Jae-Sung Lim, Minwoo Lee, Kyung-Ho Yu

**Affiliations:** 1 Division of Cardiology, Department of Internal Medicine, Dongtan Sacred Heart Hospital, Hwaseong, South Korea; 2 Department of Statistics and Actuarial Science, Soongsil University, Seoul, South Korea; 3 Department of Neurology, Hallym University Sacred Heart Hospital, Hallym University College of Medicine, Anyang, South Korea; 4 Department of Neurology, Kangdong Sacred Heart Hospital, Seoul, South Korea; 5 Department of Neurology, Chuncheon Sacred Heart Hospital, Chuncheon, South Korea; 6 Department of Neurology, Asan Medical Center, Ulsan University College of Medicine, Seoul, South Korea

**Keywords:** Neurology, Risk factors

## Abstract

We aimed to investigate the effects of exercise habit changes on the risk of incident dementia after ischemic stroke using the Korean National Health Insurance Services Database. This study included 223,426 patients with a new diagnosis of ischemic stroke between 2010 and 2016 who underwent two serial ambulatory health checkups. The participants were divided into four categories according to their habit change or regular exercise: persistent non-exercisers, new exercisers, exercise dropouts, and exercise maintainers. The primary outcome was new diagnosis of dementia. Multivariate Cox proportional models were used to assess the effects of changes in exercise habits on the risk of incident dementia. After a median of 4.02 years of follow-up, 22,554 (10.09%) dementia cases were observed. After adjusting for covariates, exercise dropouts, new exercisers, and exercise maintainers were significantly associated with a lower risk of incident dementia than persistent non-exercisers (adjusted hazard ratio [aHR] 0.937; 95% confidence interval [CI] 0.905–0.970, aHR 0.876; 95% CI 0.843–0.909, aHR 0.705; 95% CI 0.677–0.734, respectively). The impact of changes in exercise habit was more prominent in the 40–65 years age group. An energy expenditure ≥ 1000 metabolic equivalents of task-min/wk post-stroke, regardless of pre-stroke physical activity status, was mostly associated with a lower risk of each outcome. In this retrospective cohort study, initiating or continuing moderate-to-vigorous exercise after ischemic stroke was associated with a lower risk of dementia development. Further, pre-stroke regular physical activity also reduced the risk of incident dementia. The promotion of exercise in ambulatory stroke patients may reduce their future risk of incident dementia.

## Introduction

Post-stroke cognitive impairment and dementia (PSCID) is an underrepresented but disabling disease after ischemic stroke (IS), with a prevalence ranging from 25 to 83%^1–4^. PSCID not only hinders functional independence but is also associated with recurrent stroke and death^5–7^. As both stroke- and subsequent cognitive decline-related societal and individual burdens are expected to increase in the future, cognitive status after stroke requires more thorough management and preventive measures for potentially modifiable risk factors.

Among the risk factors for PSCID, a sedentary lifestyle has been suggested as one of the main modifiable factors for the prevention of PSCID. Regular, moderate-intensity exercises are not only associated with cardiovascular outcomes but are also associated with cognitive status after IS^8–10^. Although the impact of regular exercise on both stroke and cognitive impairment has been supported by numerous studies, the evidence of benefits of exercise on cognitive outcomes after stroke is scarce^11^. Furthermore, the impact of exercise habit patterns before and after IS on cognitive outcomes has not yet been addressed. Given the paucity of pharmaceutical management for the prevention of PSCID after stroke, emphasizing the optimal management of stroke and identifying alternative treatment options that can mitigate cognitive deficits, including promoting exercise habits, should be part of post-stroke care. Therefore, we aimed to investigate the effects of exercise habit change on the risk of incident dementia after IS diagnosis using the Korean National Health Insurance Services Database. In addition, we examined whether the amount of regular exercise had an impact on cognitive status after stroke.

## Methods

### Data source and study population

We used a population-based dataset from the Korean National Health Information Database (K-NHID). The K-NHID incorporates data from the Korean National Health Insurance Service (NHIS), a mandatory health insurance program that covers more than 97% of the Korean population. All insured adults are provided with biennial general health checkups, including demographic, clinical, and laboratory variables, and a self-administered questionnaire regarding lifestyle and behavior habits. Furthermore, the K-NHID consists of demographic information (including age, sex, weight, height, income, and socioeconomic status) and medical claim data (inpatient and outpatient usage of medical services, medical bills, prescription records, and date of death). This study was approved by the institutional review board of Dongtan Sacred Heart Hospital (IRB number: HDT 2022-04-002). Participants who underwent national health checkups provided written informed consent for the use of their data for research purposes. All study methods were performed in accordance with the Declaration of Helsinki.

**Figure 1:**
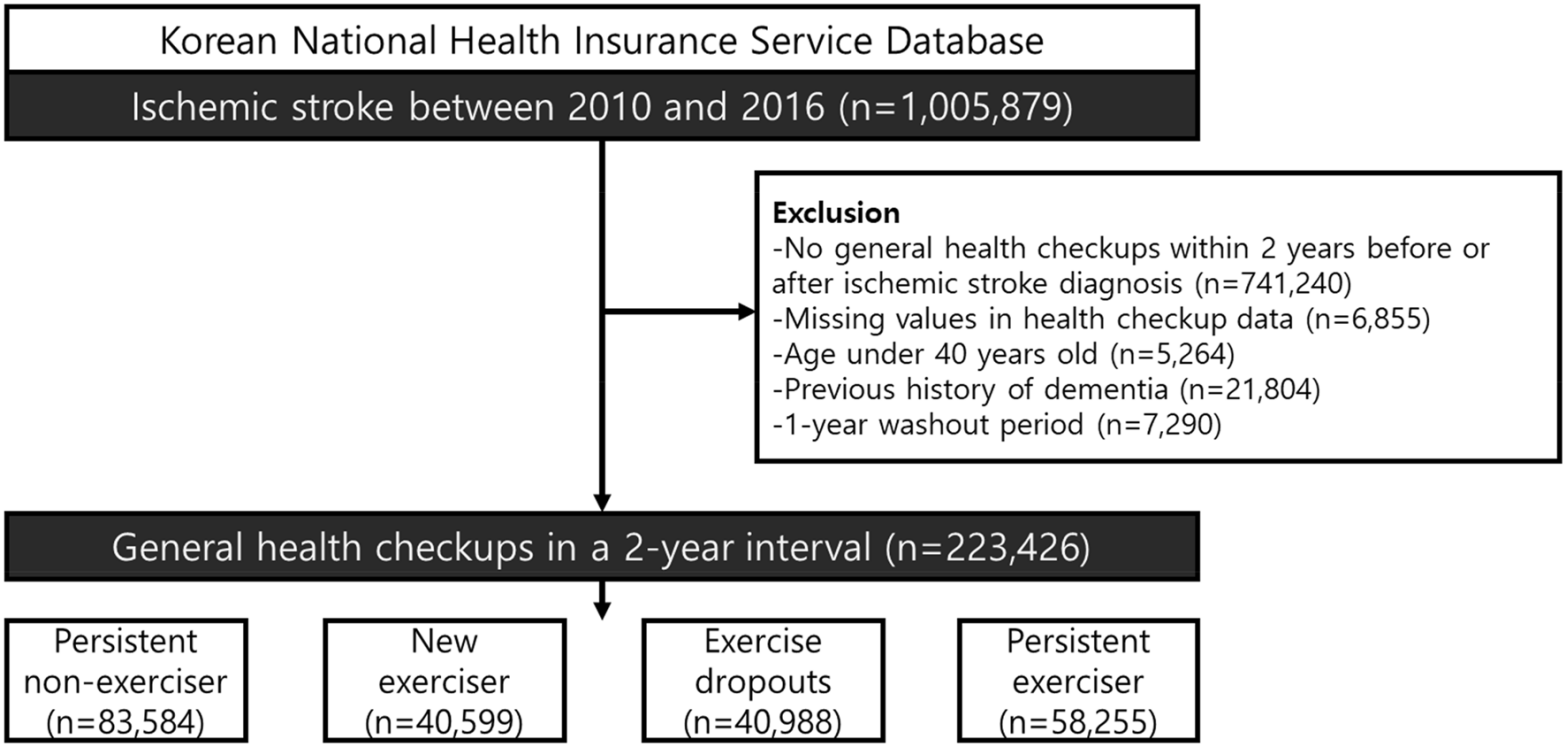
Flowchart of the selection of subjects.

We identified 1,005,879 patients who were newly diagnosed with acute IS between January 2010 and December 2016. Ischemic stroke was defined by the diagnosis of ICD-10 codes I63 and I64 with admission and combined brain imaging studies, including computed tomography (CT) or magnetic resonance image (MRI)^12^. This definition has been widely used in previous K-NHID studies and is well-validated with high accuracy, having 92.2% positive predictive value and 91.2% sensitivity^13–16^. We excluded patients who did not undergo two consecutive health checkups within two years before and after the diagnosis of IS. Among the remaining 264,639 patients, those younger than 40 years and those with a history of dementia before the index date (second health examination after IS diagnosis) were also excluded. Lastly, a 1-year washout period from the index date to remove the early occurrence of dementia additionally excluded 7290 patients. Finally, 223,426 patients with IS were included in the study (Fig. 1).

### Main exposure: exercise habit

The self-reported lifestyle behavior questionnaire, which included information on intensity and frequency of exercise, was conducted at serial health checkups before and after the diagnosis of IS. This questionnaire based on International Physical Activity Questionnaire (IPAQ), which was developed by World Health Organization and conversion to Korean version developed by Oh et al.^17^, and validated by other article^18^. The exercise section of the questionnaire consisted of three questions asking for each frequency of light, moderate, and vigorous exercise on a weekly basis during the recent weeks. Light-intensity exercise was defined as walking slowly or sweeping carpets for more than 30 min, moderate-intensity exercise was defined as bicycling leisurely, walking at a brisk pace, or playing tennis for more than 30 min, and vigorous-intensity exercise included running, climbing, bicycling quickly, or aerobics for more than 20 min. In this study, regular physical activity was defined as performing moderate or vigorous exercise at least once a week. To measure the influence of the amount of energy expenditure on cognitive outcomes, we stratified the regular physical activity groups according to energy expenditure using metabolic equivalents of tasks (METs). We rated light-, moderate-, and vigorous-intensity exercise as 2.9, 4.0, and 7.0, respectively, to calculate the energy expenditure^19^. The total energy expenditure, i.e., summation of multiplying METs by the frequency of each exercise together with the minimum duration, was stratified into < 1000 and ≥ 1000 MET-min/wk^20^.

**Figure 2:**
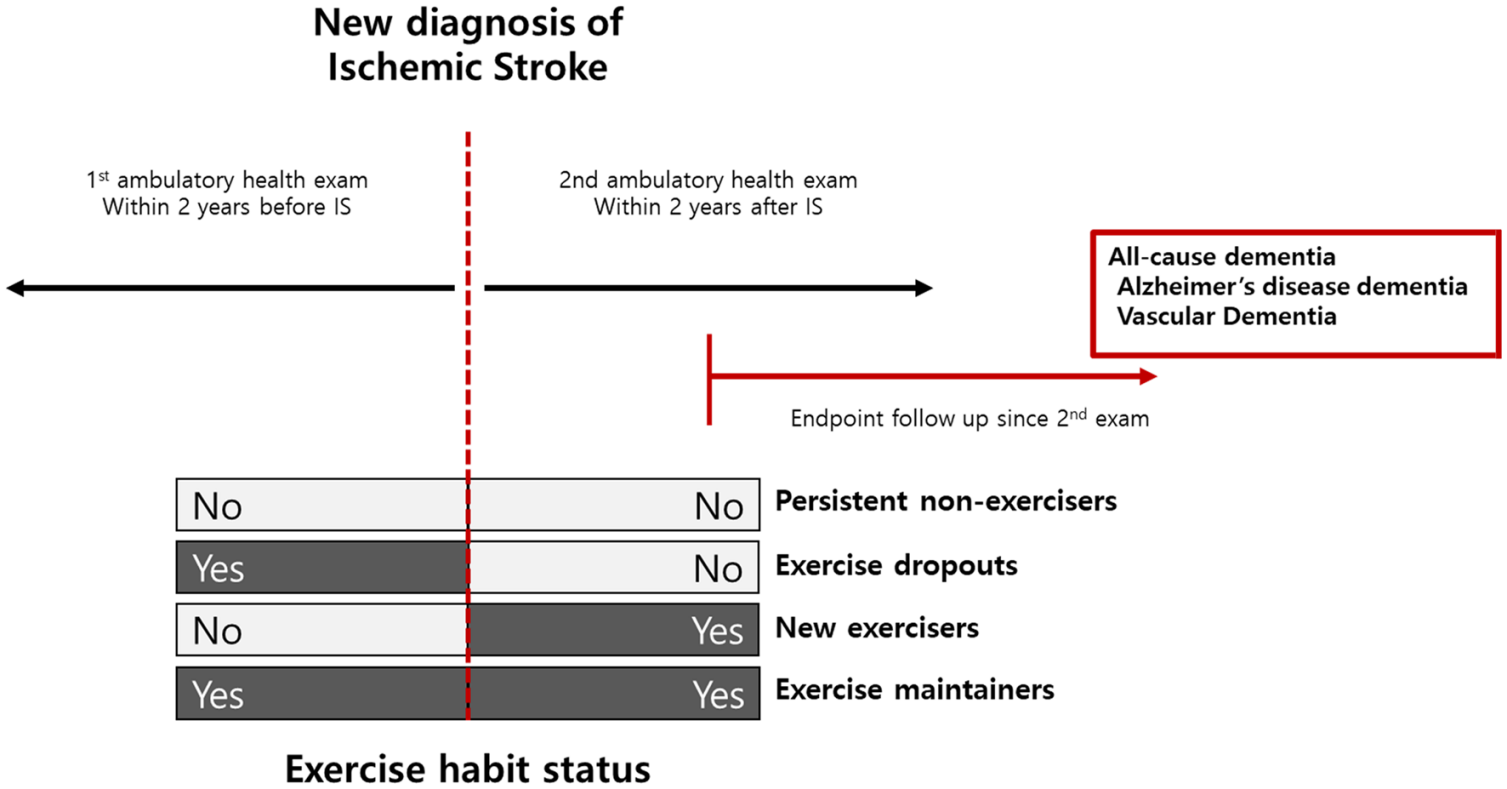
Definition of exercise habit status and the detailed study protocol.

The study participants were divided into four groups by comparing the categorization of regular physical activity status at health checkups before and after IS diagnosis: (1) persistent non-exercisers, (2) exercise dropouts, (3) new exercisers, and (4) exercise maintainers. The overall study configuration and design are shown in Fig. 2.

### Covariates and outcome definition

The primary outcome of this study was the incidence of all-cause dementia, including Alzheimer’s disease (AD), vascular dementia (VaD), and other types of dementia (ICD-10 diagnostic codes: F00, F01, F02, F03, G30, or G31). Outcome events were defined when both ICD-10 codes and the prescription records of anti-dementia drugs, including donepezil, galantamine, rivastigmine, and memantine were used. This definition has been widely accepted with high accuracy, having 94.7% positive predictive value in K-NHID^21^. The secondary outcomes included AD (F00 or G30) and VaD (F01). The date of the second health checkup was defined as the index date, and the participants were followed up until December 31, 2019 or until the development of the primary outcome, whichever came first.

We obtained information from the second general health checkup based on previous studies related to risk factors for dementia. Specifically, we obtained demographic data, including age, sex, height, body weight, and waist circumference, as well as information regarding health-related lifestyles, including smoking status, categorized as a current smoker or not, and alcohol consumption status, categorized as alcohol users (any alcohol consumption) or not. Baseline comorbidities including hypertension, dyslipidemia, diabetes mellitus, and chronic kidney disease were also obtained. The operational definition of covariates and outcomes in cardiovascular research is well documented in several studies based on K-NHID^12^. CKD was defined as an estimated glomerular filtration rate (eGFR) of < 60 mL/min/1.73 m^2^ calculated using the CKD Epidemiology Collaboration (CKD-EPI) equation. Low-income level was defined when the participants were medical benefit beneficiaries and were included in the lowest quartile of income levels. Further, laboratory data, including levels of random glucose, total cholesterol, glomerular filtration rate, and systolic/diastolic blood pressure, were also obtained at the ambulatory health exam visits after index stroke. Among these variables, age, sex, smoking status, alcohol consumption, income level, history of diabetes mellitus, hypertension, dyslipidemia, and chronic kidney disease were used as covariates.

### Statistical analysis

Descriptive statistics were used to analyze the baseline and demographic characteristics of the study participants, which were presented as the mean ± standard deviation for continuous variables and numbers with frequencies for categorical variables. The differences in demographic and clinical characteristics between the study groups are compared using one-way analysis of variance for the continuous variables and chi-square test for the categorical variables. The annual incidence of dementia was calculated by dividing the number of events by 1000 person-years (PY).

Multivariate Cox proportional hazards regression models were used to estimate the adjusted hazard ratios (aHR) with 95% confidence intervals (CIs). Persistent non-exercisers were used as the reference group in the analysis. The Cox models were sequentially adjusted as follows: Model 1 for age and sex and Model 2 for covariates of Model 1 plus all confounders, including the history of hypertension, diabetes, dyslipidemia, chronic kidney disease, alcohol consumption, smoking status, and income levels. The same regression methods were used to analyze the secondary outcomes, AD and VaD.

In addition, we conducted a subgroup analysis according to age group (40–65 years versus 65+ years) and sex. To identify whether the amount of physical activity is associated with the risk of incident dementia, we stratified the new exercisers into two subgroups by MET-min/wk with a cutoff of 1000. We performed all statistical analyses using SAS 9.4 software (SAS Institute, Cary, NC, USA) and p-values < 0.05 were considered statistically significant.

### Ethics approval and consent to participate

This study was approved by the institutional review board of Dongtan Sacred Heart Hospital (IRB number: HDT 2022-04-002). Participants who underwent national health checkups provided written informed consent for the use of their data for research purposes.

## Results

A total of 223,426 acute IS patients (mean age, 63.75 ± 10.26 years; male, 51.61%) were finally included in the study. The percentages of persistent non-exercisers, exercise dropouts, new exercisers, and persistent exercisers were 37.4%, 18.3%, 18.2%, and 26.1%, respectively. The baseline characteristics of each group are presented in Table 1. Compared to persistent exercisers, persistent non-exercisers tended to be older, female, and have a higher prevalence of diabetes, hypertension, and chronic kidney diseases.

**Table 1:** Baseline characteristics according to the exercise habit changes in patients with ischemic stroke.

	Total	Persistent non-exerciser	Exercise dropouts	New exerciser	Persistent exerciser	p-value
	(N = 223,426)	(N = 83,584)	(N = 40,988)	(N = 40,599)	(N = 58,255)	
Age at stroke onset	63.75 ± 10.26	65.98 ± 10.06	64.37 ± 10.04	62.98 ± 10.10	60.64 ± 9.96	< 0.0001
Sex, male	115,300 (51.61)	36,465 (43.63)	21,291 (51.94)	20,842 (51.34)	36,702 (63.00)	< 0.0001
Current smoker	27,621 (12.36)	10,323 (12.35)	4875 (11.89)	5114 (12.60)	7309 (12.55)	0.007
Any alcohol consumption	57,423 (25.70)	15,730 (18.82)	9362 (22.84)	10,972 (27.03)	21,359 (36.66)	< 0.0001
Low income	36,998 (16.99)	14,646 (17.98)	6816 (17.08)	7000 (17.66)	8536 (15.03)	< 0.0001
Obesity	88,303 (39.52)	33,138 (39.65)	16,164 (39.44)	16,098 (39.65)	22,903 (39.32)	0.574
Body mass index	24.31 ± 3.12	24.27 ± 3.27	24.30 ± 3.12	24.33 ± 3.08	24.36 ± 2.91	< 0.0001
Diabetes mellitus	56,148 (25.13)	22,065 (26.40)	10,646 (25.97)	10,052 (24.76)	13,385 (22.98)	< 0.0001
Hypertension	144,688 (64.76)	56,625 (67.75)	26,974 (65.81)	25,944 (63.90)	35,145 (60.33)	< 0.0001
Dyslipidemia	124,011 (55.50)	46,139 (55.20)	22,914 (55.90)	22,734 (56.00)	32,224 (55.32)	0.014
Chronic kidney disease	27,386 (12.26)	12,035 (14.40)	5250 (12.81)	4632 (11.41)	5469 (9.39)	< 0.0001
Random glucose	105.47 ± 28.75	105.99 ± 30.30	106.02 ± 29.41	104.94 ± 27.79	104.70 ± 26.56	< 0.0001
Total cholesterol	181.69 ± 41.58	182.85 ± 41.80	181.30 ± 41.78	181.31 ± 41.71	180.56 ± 40.97	< 0.0001
Glomerular filtration rate	84.33 ± 42.83	83.11 ± 37.97	83.82 ± 42.16	85.04 ± 45.10	85.94 ± 47.90	< 0.0001
Systolic blood pressure	127.12 ± 15.27	127.90 ± 15.74	127.38 ± 15.43	126.77 ± 15.07	126.07 ± 14.53	< 0.0001
Diastolic blood pressure	77.23 ± 9.84	77.24 ± 9.98	77.26 ± 9.90	77.20 ± 9.76	77.21 ± 9.64	0.789

**Figure 3:**
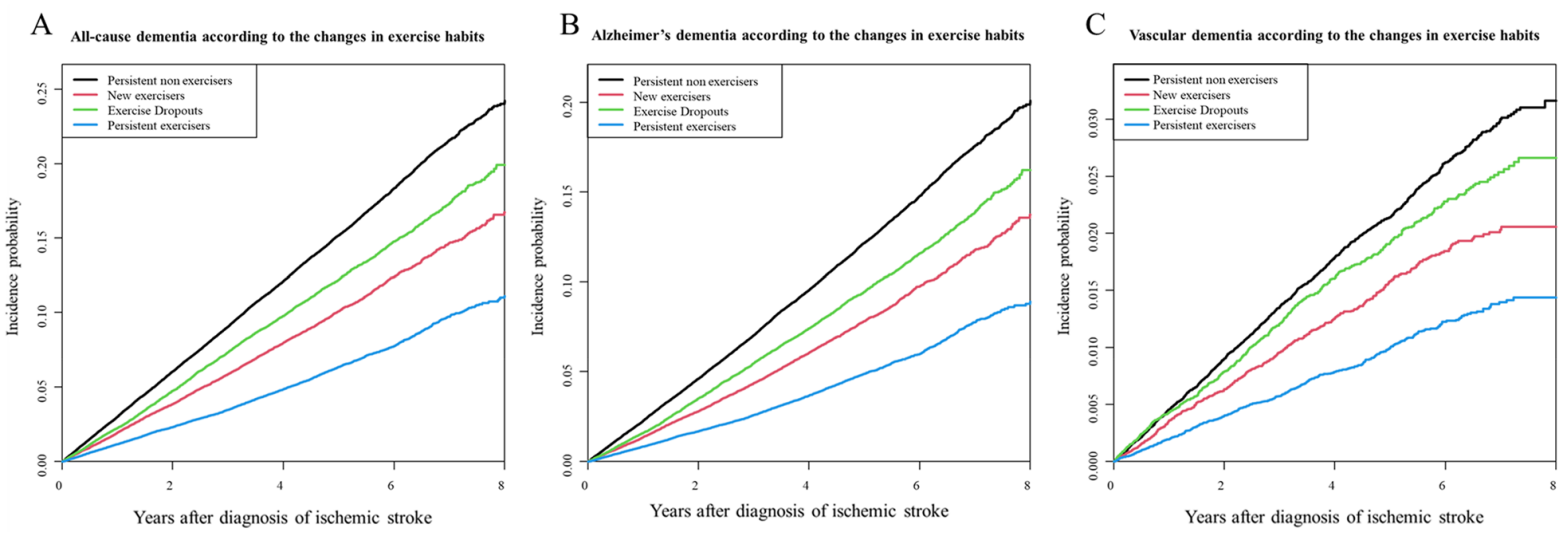
Incidence probability of all-cause dementia (**A**, p < 0.001), Alzheimer's dementia (**B**, p < 0.001), and vascular dementia (**C**, p < 0.001) after ischemic stroke according to to change of physical activity habits. *Adjusted for age, sex, alcohol consumption, smoking, income, diabetes, hypertension, dyslipidemia, and chronic kidney disease.

During a mean follow-up of 4.13 ± 2.03 years, 22,554 (10.09%) patients were newly diagnosed with all-cause dementia: 17,457 (7.81%) with AD and 3,146 (1.4%) with VaD. Overall, new exercisers and persistent exercisers had a lower risk of all-cause dementia (aHR 0.876; 95% CI 0.843–0.909, aHR 0.705; 95% CI 0.677–0.734, respectively), AD, and VaD than persistent non-exercisers, after adjusting for covariates. Exercise dropouts also had a lower risk of developing all-cause dementia (aHR 0.937; 95% CI 0.905–0.970) and AD. The Kaplan–Meier curves, crude event numbers, incidence rates (IR), and aHRs for the outcomes are presented in Fig. 3 and Table 2.

**Table 2:** Risk for all-cause dementia, Alzheimer’s disease and vascular dementia according to the exercise habit changes.

	Number of patients	Number of events	Duration (PY)	IR (per 1000PY)	aHR* (95% CI) Model 1	aHR* (95% CI) Model 2
All-cause dementia						
Persistent non-exerciser	83,584	11,189	337,006	33.20	1 (Ref.)	1 (Ref.)
Exercise dropouts	40,988	4434	170,773	26.27	0.930 (0.898, 0.963)	0.937 (0.905, 0.970)
New exerciser	40,599	3673	168,779	21.51	0.864 (0.833, 0.898)	0.876 (0.843, 0.909)
Persistent exerciser	58,255	3258	245,512	13.27	0.684 (0.657, 0.712)	0.705 (0.677, 0.734)
Alzheimer’s dementia						
Persistent non-exerciser	83,584	8769	337,006	26.02	1 (Ref.)	1 (Ref.)
Exercise dropouts	40,988	3384	170,773	20.05	0.920 (0.884, 0.957)	0.927 (0.891, 0.965)
New exerciser	40,599	2811	168,779	16.46	0.863 (0.826, 0.900)	0.873 (0.837, 0.912)
Persistent exerciser	58,255	2493	245,512	10.15	0.699 (0.667, 0.731)	0.720 (0.687, 0.753)
Vascular dementia						
Persistent non-exerciser	83,584	1483	337,006	4.40	1 (Ref.)	1 (Ref.)
Exercise dropouts	40,988	650	170,773	3.85	0.964 (0.879, 1.058)	0.972 (0.886, 1.066)
New exerciser	40,599	525	168,779	3.07	0.852 (0.771, 0.942)	0.867 (0.784, 0.958)
Persistent exerciser	58,255	488	245,512	1.99	0.642 (0.578, 0.714)	0.668 (0.601, 0.742)

IR, Incidence rate; aHR, adjusted hazard ratio; PY, person-years.

^#^Model 1: age and sex-adjusted, Model 2: Model 1 + smoking status, alcohol status, low income, history of diabetes, hypertension, dyslipidemia and chronic kidney disease adjusted.

We performed a subgroup analysis to determine whether the association between changes in exercise habits and the risk of dementia was affected by age or sex. No significant interaction was observed between sex and changes in exercise habits on the risk of all-cause dementia, AD, and VaD. Consistent findings were observed in both age subgroups as in the primary analysis. However, significant interactions were observed between age and changes in exercise habits on the risk of incident all-cause dementia and AD. The beneficial effects of persistent or initiated exercise habits were more prominent in the younger patient group (< 65 years, p < 0.001 for all-cause dementia and p < 0.005 for AD, Table 3).

**Table 3:** Subgroup analyses according to the age and sex group and risk for all-cause dementia.

	Exercise habit change status	Number of patients	Number of events of all-cause dementia	Duration (PY)	IR (per 1000PY)	aHR* (95% CI) Model 1	aHR* (95% CI) Model 2
Male	Persistent non-exerciser	36,465	4512	142,795	31.60	1 (Ref.)	1 (Ref.)
	Exercise dropouts	21,291	2220	86,315	25.96	0.924 (0.878, 0.972)	0.930 (0.884, 0.979)
	New exerciser	20,842	1770	85,529	20.51	0.840 (0.795, 0.887)	0.851 (0.806, 0.900)
	Persistent exerciser	36,702	1980	154,198	12.84	0.665 (0.631, 0.702)	0.687 (0.652, 0.725)
Female	Persistent non-exerciser	47,119	6677	194,211	34.38	1 (Ref.)	1 (Ref.)
	Exercise dropouts	19,697	2214	84,458	26.59	0.932 (0.889, 0.978)	0.941 (0.896, 0.987)
	New exerciser	19,757	1903	83,250	22.53	0.886 (0.842, 0.932)	0.896 (0.852, 0.943)
	Persistent exerciser	21,553	1278	91,314	14.00	0.707 (0.666, 0.751)	0.727 (0.684, 0.772)
p for interaction						0.316	0.384
40–64 years	Persistent non-exerciser	32,993	1111	144,916	7.67	1 (Ref.)	1 (Ref.)
	Exercise dropouts	18,717	545	93,060	6.61	0.889 (0.802, 0.985)	0.904 (0.816, 1.002)
	New exerciser	20,891	537	82,452	5.77	0.805 (0.726, 0.893)	0.824 (0.743, 0.913)
	Persistent exerciser	35,485	568	155,336	3.66	0.554 (0.501, 0.613)	0.580 (0.524, 0.642)
≥ 65 years	Persistent non-exerciser	50,591	10,078	192,090	52.46	1 (Ref.)	1 (Ref.)
	Exercise dropouts	22,271	3889	77,713	45.05	0.930 (0.896, 0.965)	0.936 (0.902, 0.971)
	New exerciser	19,708	3136	86,326	40.35	0.868 (0.834, 0.904)	0.878 (0.844, 0.915)
	Persistent exerciser	22,770	2690	90,175	29.83	0.710 (0.680, 0.741)	0.730 (0.699, 0.762)
p for interaction						< 0.001	< 0.001

IR, Incidence rate; aHR, adjusted hazard ratio; PY, person-years.

^#^Model 1: age and sex adjusted, Model 2: Model 1 + smoking status, alcohol status, low income, history of diabetes, hypertension, dyslipidemia and chronic kidney disease adjusted.

Multivariable Cox proportional hazards analysis for the risk of dementia according to the stratified amount of exercise with reference to persistent non-exercisers is presented in Supplemental Table 1. Overall, regardless of pre-stroke exercise habits, exercising ≥ 1000 MET-min/wk post stroke was generally associated with a lower risk of all-cause dementia, AD, and VaD than exercising < 1000 MET-min/wk. However, exercising ≥ 1000 MET-min/wk post stroke was not associated with a lower risk of VaD compared to exercising < 1000 MET-min/wk post-stroke in patients who did not regularly exercise before the index stroke.

## Discussion

In this nationwide, population-based, cohort study, we revealed the following principal findings. First, both the initiation and maintenance of regular exercise after IS diagnosis were significantly associated with a lower risk of incident dementia. Exercise dropout was also associated with a lower risk of dementia, although the relative risk reduction rate was low. Second, performing exercise at an energy expenditure level of ≥ 1000 MET-min/wk was associated with significantly lower risks of incident dementia compared to mild intensity exercise, except in the association between new exercisers and vascular dementia.

IS is associated with an increased risk of PSCID, which is a major burden on stroke survivors. While most of the studies on IS focused on cardiovascular outcomes, including recurrent stroke or vascular death, PSCID deserves more attention, as the impact of cognitive status on the quality of life after stroke is substantial^22^. Clinically, no effective medications have been developed for the prevention of cognitive deficits after stroke. Furthermore, prescription of anti-dementia drugs, including donepezil, after the diagnosis of post-stroke dementia is rarely recommended with little evidence of their effectiveness in the current guidelines^23^. Thus, determining modifiable risk factors that are valid and easily accessible to the post-stroke population is essential.

Recently, cumulative research has shown that regular physical activity is a potentially effective modality that may reduce cognitive deficits in both the general aging population^24^ and the stroke population. Additionally, meta-analyses of 14 and 22 randomized clinical trials have revealed that structured physical training has a significant positive effect on cognition after stroke with small-to-moderate cognitive gains^25,26^. While most observational studies and randomized clinical trials have focused on post-stroke physical activity status, our results provide novel evidence that maintaining or initiating physical activity is associated with a lower risk of dementia with relative risk reduction of 13.4–29.5%. Furthermore, pre-stroke exercise was also associated with a lower risk of dementia development, even if the patients stopped regular physical activities after the stroke, although the relative risk reduction was small. This finding suggests that a healthy pre-stroke lifestyle with regular physical activity may continuously reduce the risk of dementia after stroke. While both mild (MET-min/wk < 1000) and moderate-to-intense (MET-min/wk ≥ 1000) physical activity were associated with a reduced risk of dementia, regardless of the pre-stroke physical activity status, moderate-to-intense physical activity showed a trend of a reduced risk of dementia. These results are consistent with previous literature showing that moderate-intensity exercises showed a greater degree of improvement of cognitive function, while low-intensity physical activity did not improve cognitive ability^26^.

In the subgroup analysis, the risk reduction was more prominent in the younger age group. This finding is most likely related to the potential mechanisms underlying the beneficial effects of physical activity on cognitive outcomes. Previous in vivo studies have revealed that aerobic exercise after stroke increases the levels of brain-derived neutronic factors, synaptogenesis, dendritic branching^27^, and neurogenesis^28^, which eventually improve cognitive function. These physical activity-related neuronal changes are most remarkable in young rodents^29^. Thus, the relatively lower risk reduction in the older group may be attributed to their decreased neuroplasticity or capacity for neurogenesis. Therefore, our results indicate that regular exercise should be encouraged, even in younger stroke survivors with a reservoir for cognitive recovery.

Our study population only included ambulatory stroke survivors who could attend ambulatory health checkups after the index stroke. The health checkup program is run by the Korean NHIS, and patients must attend the designated hospital for examination. Thus, patients who were hospitalized or had severe motor deficits or aphasia that hindered them from attending the designated hospital could not be included in this study. As a result, our study results were mainly confined to patients with mild IS. A previous meta-analysis showed that those with mild motor deficits who could participate in structured physical activity programs also achieved a moderate amount of cognitive gains^25^. Thus, our study is consistent with previous studies that indicate that post-stroke ambulatory survivors who can actively participate in physical activity may be at reduced risk of incident dementia.

This study has several limitations. First, given the nature of population-based cohort studies based on claims data, the stroke population data lacked important clinical variables, including education level, pre-stroke cognitive status, stroke severity, discharge antithrombotics, discharge statins, presence of depression or other mood disorders,and history of atrial fibrillation, which are established risk factors of dementia after stroke. Second, potential changes in exercise habit status from the index date to the end of the follow-up period might have induced bias. However, as most of the changes in exercise habits in the stroke population would be the dropout of exercise, the potential bias would have underestimated the effects of initiation of continuing exercise in our study. Third, exercise habit assessment was solely conducted according to the patient’s self-reporting, with a limited quality of questions and may not reflect the actual exercise level. Furthermore, the timing of exercise initiation after acute stroke was not available. Finally, as our study population only included those who were capable of visiting ambulatory national health checkups and perform self-reported questionnaires, we can speculate that the study population had IS of mild severity. Thus, our findings may not be generalized to all stroke population. Despite these limitations, this large, nationwide cohort study with structured questionnaires enabled us to determine the effects of changes in exercise habits before and after stroke diagnosis on incident dementia using real-world data. We provided novel evidence by collecting the level of physical activity before and after stroke diagnosis, which differentiates this study from the previous literature.

## Conclusion

In this nationwide, population-based, retrospective cohort study, initiating or maintaining regular exercise after IS diagnosis was associated with a lower risk of incident dementia compared to persistent non-exercisers. Additionally, regular exercisers who failed to maintain exercise habits after IS also had decreased risk for dementia. Regular moderate exercise of ≥ 1000 MET-min/wk might be optimal for deriving maximal benefits on cognitive outcomes after stroke. Thus, exercise encouragement in ambulatory stroke patients is warranted.

## Supplementary Information

Supplementary Table 1.

## Data Availability

The anonymized dataset for this study is publicly available from the Korean National Health Insurance Sharing Service and can be accessed at https://nhiss.nhis.or.kr/bd/ab/bdaba000eng.do.

## Abbreviations

IS: Ischemic stroke
PSCID: Post-stroke cognitive impairment and dementia
MET: Metabolic equivalent of tasks
AD: Alzheimer’s disease
VaD: Vascular dementia
aHR: Adjusted hazard ratio
