# Supplementary material for: Association between exercise habit changes and incident dementia after ischemic stroke
Source: Sci Rep. 2023 Mar 9;13:3959. doi: 10.1038/s41598-023-31229-z (PMC9998861; doi:10.1038/s41598-023-31229-z)
Supplement: Supplementary file 1 — Supplementary Table 1. [file 41598_2023_31229_MOESM1_ESM.docx]

| **Supplemental Table 1. Subgroup analyses according to the pre- and post-stroke MET-min/wk** | | | | | | | |
| --- | --- | --- | --- | --- | --- | --- | --- |
| Pre MET-min/wk | Post MET-min/wk | Number of patients | Number of events | Duration  (PY) | IR  (per 1,000PY) | aHR*(95% CI) Model 1 | aHR*(95% CI) Model 2 |
| **Any Dementia** |  |  |  |  |  |  |  |
| Non-exerciser | Non-exerciser | 83,584 | 11,189 | 337,006 | 33.20 | 1(Ref.) | 1(Ref.) |
|  | MET-min/wk <1,000 | 22,906 | 2,030 | 96,529 | 21.03 | 0.865(0.825,0.907) | 0.879(0.838,0.921) |
|  | MET-min/wk ≥1,000 | 17,693 | 1,643 | 74,244 | 22.13 | 0.864(0.820,0.910) | 0.872(0.828,0.918) |
| MET-min/wk <1,000 | Non-exerciser | 23,631 | 2,436 | 97,974 | 24.86 | 0.924(0.884,0.965) | 0.933(0.893,0.975) |
|  | MET-min/wk <1,000 | 18,226 | 867 | 77,429 | 11.20 | 0.677(0.632,0.726) | 0.704(0.657,0.755) |
|  | MET-min/wk ≥1,000 | 12,095 | 632 | 51,551 | 12.26 | 0.657(0.606,0.712) | 0.675(0.623,0.732) |
| MET-min/wk ≥1,000 | Non-exerciser | 17,357 | 1,998 | 70,805 | 28.22 | 0.938(0.894,0.983) | 0.942(0.898,0.988) |
|  | MET-min/wk <1,000 | 10,353 | 696 | 43,841 | 15.88 | 0.749(0.694,0.809) | 0.767(0.710,0.828) |
|  | MET-min/wk ≥1,000 | 17,581 | 1,063 | 72,689 | 14.62 | 0.668(0.627,0.712) | 0.688(0.646,0.733) |
| **Alzheimer’s Dementia** | |  |  |  |  |  |  |
| Non-exerciser | Non-exerciser | 83,584 | 8,769 | 337,006 | 26.02 | 1(Ref.) | 1(Ref.) |
|  | MET-min/wk <1,000 | 22,906 | 1,571 | 96,529 | 16.27 | 0.869(0.824,0.917) | 0.882(0.836,0.931) |
|  | MET-min/wk ≥1,000 | 17,693 | 1,240 | 74,244 | 16.70 | 0.854(0.804,0.907) | 0.862(0.812,0.915) |
| MET-min/wk <1,000 | Non-exerciser | 23,631 | 1,859 | 97,974 | 18.97 | 0.911(0.867,0.958) | 0.920(0.875,0.968) |
|  | MET-min/wk <1,000 | 18,226 | 665 | 77,429 | 8.59 | 0.693(0.640,0.751) | 0.719(0.664,0.779) |
|  | MET-min/wk ≥1,000 | 12,095 | 494 | 51,551 | 9.58 | 0.687(0.627,0.753) | 0.706(0.644,0.773) |
| MET-min/wk ≥1,000 | Non-exerciser | 17,357 | 1,525 | 70,805 | 21.54 | 0.931(0.881,0.983) | 0.936(0.886,0.988) |
|  | MET-min/wk <1,000 | 10,353 | 531 | 43,841 | 12.11 | 0.759(0.695,0.828) | 0.776(0.710,0.847) |
|  | MET-min/wk ≥1,000 | 17,581 | 803 | 72,689 | 11.05 | 0.674(0.627,0.726) | 0.695(0.646,0.748) |
| **Vascular Dementia** | |  |  |  |  |  |  |
| Non-exerciser | Non-exerciser | 83,584 | 1,483 | 337,006 | 4.40 | 1(Ref.) | 1(Ref.) |
|  | MET-min/wk <1,000 | 22,906 | 283 | 96,529 | 2.93 | 0.841(0.741,0.956) | 0.862(0.759,0.979) |
|  | MET-min/wk ≥1,000 | 17,693 | 242 | 74,244 | 3.26 | 0.865(0.754,0.991) | 0.872(0.761,1.000) |
| MET-min/wk <1,000 | Non-exerciser | 23,631 | 360 | 97,974 | 3.67 | 0.971(0.865,1.090) | 0.983(0.876,1.103) |
|  | MET-min/wk <1,000 | 18,226 | 126 | 77,429 | 1.63 | 0.609(0.506,0.731) | 0.642(0.534,0.771) |
|  | MET-min/wk ≥1,000 | 12,095 | 87 | 51,551 | 1.69 | 0.562(0.452,0.698) | 0.580(0.467,0.721) |
| MET-min/wk ≥1,000 | Non-exerciser | 17,357 | 290 | 70,805 | 4.10 | 0.956(0.843,1.085) | 0.958(0.844,1.087) |
|  | MET-min/wk <1,000 | 10,353 | 103 | 43,841 | 2.35 | 0.713(0.583,0.871) | 0.733(0.599,0.896) |
|  | MET-min/wk ≥1,000 | 17,581 | 172 | 72,689 | 2.37 | 0.679(0.579,0.796) | 0.704(0.600,0.826) |
| *Abbreviation: MET, metabolic equivalents of tasks | | | | | | | |
